# Supplementary material for: Cytogenotoxic potential and toxicity in adult Danio rerio (zebrafish) exposed to chloramine T
Source: PeerJ. 2023 Dec 4;11:e16452. doi: 10.7717/peerj.16452 (PMC10702335; doi:10.7717/peerj.16452)
Supplement: Supplemental Information 1 [file peerj-11-16452-s001.pdf]

Table Analyzed	histo GILL					
Data sets analyzed	A-D					
ANOVA summary						
F	7.648					
P value	0.0013					
P value summary	**					
Significant diff. among means (P < 0.05)?	Yes					
R square	0.5343					
Brown-Forsythe test						
F (DFn, DFd)	4.842 (3, 20)					
P value	0.0108					
P value summary	*					
Are SDs significantly different (P < 0.05)?	Yes					
Bartlett's test						
Bartlett's statistic (corrected)	12.51					
P value	0.0058					
P value summary	**					
Are SDs significantly different (P < 0.05)?	Yes					
ANOVA table	SS	DF	MS	F (DFn, DF	P value	
Treatment (between columns)	260.4	3	86.79	F (3, 20) =	P=0.0013	
Residual (within columns)	227	20	11.35			
Total	487.3	23				
Data summary						
Number of treatments (columns)	4					
Number of values (total)	24					
Number of families	1					
Number of comparisons per family	6					
Alpha	0.05					
Tukey's multiple comparisons test	Mean Diff.	95.00% CI	Significant?	Summary	Adjusted P	
control vs. 70	-7.458	-12.55 to -2	Yes	**	0.0029	
control vs. 140	-5.583	-11.67 to 0.	No	ns	0.0794	
control vs. 200	-8.333	-13.78 to -2	Yes	**	0.0019	
70 vs. 140	1.875	-3.899 to 7.	No	ns	0.8003	
70 vs. 200	-0.875	-5.967 to 4.	No	ns	0.9624	
140 vs. 200	-2.75	-8.836 to 3.	No	ns	0.5948	
Test details	Mean 1	Mean 2	Mean Diff.	SE of diff.	n1	
control vs. 70	0.6667	8.125	-7.458	1.819	6	
control vs. 140	0.6667	6.25	-5.583	2.174	6	
control vs. 200	0.6667	9	-8.333	1.945	6	
70 vs. 140	8.125	6.25	1.875	2.063	8	

70 vs. 200	8.125	9	-0.875	1.819	8
140 vs. 200	6.25	9	-2.75	2.174	4

Value
A-B
A-C
A-D
B-C
B-D
C-D

n2	q	DF	
8	5.798		20
4	3.631		20
6	6.059		20
4	1.285		20

6	0.6802	20
6	1.789	20

Table Analyzed	histo LIVER				
Data sets analyzed	A-D				
ANOVA summary					
F	32.15				
P value	<0.0001				
P value summary	****				
Significant diff. among means (P < 0.05)?	Yes				
R square	0.7813				
Brown-Forsythe test					
F (DFn, DFd)	1.867 (3, 27)				
P value	0.1591				
P value summary	ns				
Are SDs significantly different (P < 0.05)?	No				
Bartlett's test					
Bartlett's statistic (corrected)	31.64				
P value	<0.0001				
P value summary	****				
Are SDs significantly different (P < 0.05)?	Yes				
ANOVA table	SS	DF	MS	F (DFn, DF	P value
Treatment (between columns)	516.7	3	172.2	F (3, 27) =	P<0.0001
Residual (within columns)	144.7	27	5.358		
Total	661.4	30			
Data summary					
Number of treatments (columns)	4				
Number of values (total)	31				
Number of families	1				
Number of comparisons per family	6				
Alpha	0.05				
Tukey's multiple comparisons test	Mean Diff.	95.00% CI	Significant?	Summary	Adjusted P
control vs. 70	-5.639	-8.717 to -2	Yes	***	0.0002
control vs. 140	-7.603	-10.80 to -4	Yes	****	<0.0001
control vs. 200	-11.03	-14.22 to -7	Yes	****	<0.0001
70 vs. 140	-1.964	-5.243 to 1.	No	ns	0.3743
70 vs. 200	-5.393	-8.671 to -2	Yes	***	0.0006
140 vs. 200	-3.429	-6.815 to -0	Yes	*	0.0464
Test details	Mean 1	Mean 2	Mean Diff.	SE of diff.	n1
control vs. 70	0.1111	5.75	-5.639	1.125	9
control vs. 140	0.1111	7.714	-7.603	1.167	9
control vs. 200	0.1111	11.14	-11.03	1.167	9

70 vs. 140	5.75	7.714	-1.964	1.198	8
70 vs. 200	5.75	11.14	-5.393	1.198	8
140 vs. 200	7.714	11.14	-3.429	1.237	7

Value
A-B
A-C
A-D
B-C
B-D
C-D

n2	q	DF
8	7.09	27
7	9.217	27
7	13.37	27

7	2.319	27
7	6.366	27
7	3.919	27

Table Analyzed	AChE adults				
Data sets analyzed	A-D				
ANOVA summary					
F	0.4979				
P value	0.6906				
P value summary	ns				
Significant diff. among means (P < 0.05)?	No				
R square	0.1107				
Brown-Forsythe test					
F (DFn, DFd)	2.004 (3, 12)				
P value	0.1672				
P value summary	ns				
Are SDs significantly different (P < 0.05)?	No				
Bartlett's test					
Bartlett's statistic (corrected)	3				
P value	0.3916				
P value summary	ns				
Are SDs significantly different (P < 0.05)?	No				
ANOVA table	SS	DF			
Treatment (between columns)	1064521	3			
Residual (within columns)	8551773	12			
Total	9616294	15			
Data summary					
Number of treatments (columns)	4				
Number of values (total)	16				
Number of families	1				
Number of comparisons per family	6				
Alpha	0.05				
Tukey's multiple comparisons test	Mean Diff.	95.00% CI of diff.	Significant?	Summary	Adjusted P
control vs. 1	665.8	-1106 to 2438	No	ns	0.6875
control vs. 2	423.3	-1349 to 2195	No	ns	0.8916
control vs. 3	134.8	-1637 to 1907	No	ns	0.9957
1 vs. 2	-242.5	-2015 to 1530	No	ns	0.9763
1 vs. 3	-531	-2303 to 1241	No	ns	0.8104
2 vs. 3	-288.5	-2061 to 1484	No	ns	0.9614
Test details	Mean 1	Mean 2	Mean Diff.	SE of diff.	n1
control vs. 1	2723	2057	665.8	596.9	4
control vs. 2	2723	2299	423.3	596.9	4
control vs. 3	2723	2588	134.8	596.9	4
1 vs. 2	2057	2299	-242.5	596.9	4
1 vs. 3	2057	2588	-531	596.9	4
2 vs. 3	2299	2588	-288.5	596.9	4

Value
A-B
A-C
A-D
B-C
B-D
C-D

n2	q	DF
4	1.577	12
4	1.003	12
4	0.3192	12
4	0.5745	12
4	1.258	12
4	0.6835	12

MS	F (DFn, DF	P value
354840	F (3, 12) =	P=0.6906
712648		

Table Analyzed	MN adultos				
Data sets analyzed	A-D				
ANOVA summary					
F	1.797				
P value	0.1689				
P value summary	ns				
Significant diff. among means (P < 0.05)?	No				
R square	0.1524				
Brown-Forsythe test					
F (DFn, DFd)	1.797 (3, 30)				
P value	0.1689				
P value summary	ns				
Are SDs significantly different (P < 0.05)?	No				
Bartlett's test					
Bartlett's statistic (corrected)					
P value					
P value summary					
Are SDs significantly different (P < 0.05)?					
ANOVA table	SS	DF	MS	F (DFn, DF	P value
Treatment (between columns)	1.792	3	0.5975	F (3, 30) =	P=0.1689
Residual (within columns)	9.972	30	0.3324		
Total	11.76	33			
Data summary					
Number of treatments (columns)	4				
Number of values (total)	34				
Number of families	1				
Number of comparisons per family	6				
Alpha	0.05				
Tukey's multiple comparisons test	Mean Diff.	95.00% CI	Significant?	Summary	Adjusted P
control vs. 70	-0.4444	-1.183 to 0.	No	ns	0.3749
control vs. 140	-0.375	-1.137 to 0.	No	ns	0.5464
control vs. 200	-0.625	-1.387 to 0.	No	ns	0.138
70 vs. 140	0.06944	-0.6923 to (	No	ns	0.9945
70 vs. 200	-0.1806	-0.9423 to (	No	ns	0.9166
140 vs. 200	-0.25	-1.034 to 0.	No	ns	0.8216
Test details	Mean 1	Mean 2	Mean Diff.	SE of diff.	n1
control vs. 70	0	0.4444	-0.4444	0.2718	9
control vs. 140	0	0.375	-0.375	0.2802	9
control vs. 200	0	0.625	-0.625	0.2802	9
70 vs. 140	0.4444	0.375	0.06944	0.2802	9

70 vs. 200	0.4444	0.625	-0.1806	0.2802	9
140 vs. 200	0.375	0.625	-0.25	0.2883	8

Table Analyzed	MN adultos
Column B	70
vs.	vs.
Column A	control
Mann Whitney test	
P value	0.0824
Exact or approximate P value?	Exact
P value summary	ns
Significantly different (P < 0.05)?	No
One- or two-tailed P value?	Two-tailed
Sum of ranks in column A,B	67.50 , 103.5
Mann-Whitney U	22.5
Difference between medians	
Median of column A	0.000, n=9
Median of column B	0.000, n=9
Difference: Actual	0
Difference: Hodges-Lehmann	0

Table Analyzed	MN adultos
Column C	140
vs.	vs.
Column A	control
Mann Whitney test	
P value	0.0824
Exact or approximate P value?	Exact
P value summary	ns
Significantly different (P < 0.05)?	No
One- or two-tailed P value?	Two-tailed
Sum of ranks in column A,C	67.50 , 85.50
Mann-Whitney U	22.5
Difference between medians	
Median of column A	0.000, n=9
Median of column C	0.000, n=8
Difference: Actual	0
Difference: Hodges-Lehmann	0

Table Analyzed	MN adultos
Column D	200
vs.	vs.

Value

A-B
A-C
A-D
B-C
B-D
C-D

n2	q	DF
9	2.313	30
8	1.893	30
8	3.155	30
8	0.3506	30

8	0.9114	30	Column A	control
8	1.226	30		
			Mann Whitney test	
			P value	0.0824
			Exact or approximate P value?	Exact
			P value summary	ns
			Significantly different (P < 0.05)?	No
			One- or two-tailed P value?	Two-tailed
			Sum of ranks in column A,D	67.50 , 85.50
			Mann-Whitney U	22.5
			Difference between medians	
			Median of column A	0.000, n=9
			Median of column D	0.000, n=8
			Difference: Actual	0
			Difference: Hodges-Lehmann	0

Table Analyzed	AN adultos
Data sets analyzed	A-D
ANOVA summary	
F	4.021
P value	0.0162
P value summary	*
Significant diff. among means (P < 0.05)?	Yes
R square	0.2868
Brown-Forsythe test	
F (DFn, DFd)	2.495 (3, 30)
P value	0.0789
P value summary	ns
Are SDs significantly different (P < 0.05)?	No
Bartlett's test	
Bartlett's statistic (corrected)	7.628
P value	0.0544
P value summary	ns
Are SDs significantly different (P < 0.05)?	No

ANOVA table	SS	DF	MS	F (DFn, DF	P value
Treatment (between columns)	1.545	3	0.5149	F (3, 30) =	P=0.0162
Residual (within columns)	3.842	30	0.1281		
Total	5.386	33			

Data summary	
Number of treatments (columns)	4
Number of values (total)	34
Number of families	1
Number of comparisons per family	6
Alpha	0.05

Tukey's multiple comparisons test	Mean Diff.	95.00% CI	Significant?	Summary	Adjusted P
control vs. 70	-0.2	-0.6587 to (	No	ns	0.6404
control vs. 140	-0.1139	-0.5867 to (	No	ns	0.913
control vs. 200	-0.5764	-1.049 to -(	Yes	*	0.0122
70 vs. 140	0.08611	-0.3867 to (	No	ns	0.9595
70 vs. 200	-0.3764	-0.8492 to (	No	ns	0.1564
140 vs. 200	-0.4625	-0.9490 to (	No	ns	0.0671

Test details	Mean 1	Mean 2	Mean Diff.	SE of diff.	n1
control vs. 70	0.2111	0.4111	-0.2	0.1687	9
control vs. 140	0.2111	0.325	-0.1139	0.1739	9
control vs. 200	0.2111	0.7875	-0.5764	0.1739	9
70 vs. 140	0.4111	0.325	0.08611	0.1739	9

70 vs. 200	0.4111	0.7875	-0.3764	0.1739	9
140 vs. 200	0.325	0.7875	-0.4625	0.1789	8

Table Analyzed	AN adultos
Column B	70
vs.	vs.
Column A	control
Mann Whitney test	
P value	0.0966
Exact or approximate P value?	Exact
P value summary	ns
Significantly different (P < 0.05)?	No
One- or two-tailed P value?	Two-tailed
Sum of ranks in column A,B	66.50 , 104.5
Mann-Whitney U	21.5
Difference between medians	
Median of column A	0.2000, n=9
Median of column B	0.4000, n=9
Difference: Actual	0.2
Difference: Hodges-Lehmann	0.2

Table Analyzed	AN adultos
Column C	140
vs.	vs.
Column A	control
Mann Whitney test	
P value	0.4768
Exact or approximate P value?	Exact
P value summary	ns
Significantly different (P < 0.05)?	No
One- or two-tailed P value?	Two-tailed
Sum of ranks in column A,C	73 , 80
Mann-Whitney U	28
Difference between medians	
Median of column A	0.2000, n=9
Median of column C	0.2500, n=8
Difference: Actual	0.05
Difference: Hodges-Lehmann	0.1

Value
A-B
A-C
A-D
B-C
B-D
C-D

n2	q	DF
9	1.677	30
8	0.9263	30
8	4.688	30
8	0.7004	30

Table Analyzed	AN adultos

8	3.061	30
8	3.656	30

Column D	200
vs.	vs.
Column A	control
Mann Whitney test	
P value	0.0101
Exact or approximate P value?	Exact
P value summary	*
Significantly different (P < 0.05)?	Yes
One- or two-tailed P value?	Two-tailed
Sum of ranks in column A,D	55 , 98
Mann-Whitney U	10
Difference between medians	
Median of column A	0.2000, n=9
Median of column D	0.6500, n=8
Difference: Actual	0.45
Difference: Hodges-Lehmann	0.4
