# Supplementary figures and images for: Cytogenotoxic potential and toxicity in adult Danio rerio (zebrafish) exposed to chloramine T
Source: PeerJ. 2023 Dec 4;11:e16452. doi: 10.7717/peerj.16452 (PMC10702335; doi:10.7717/peerj.16452)

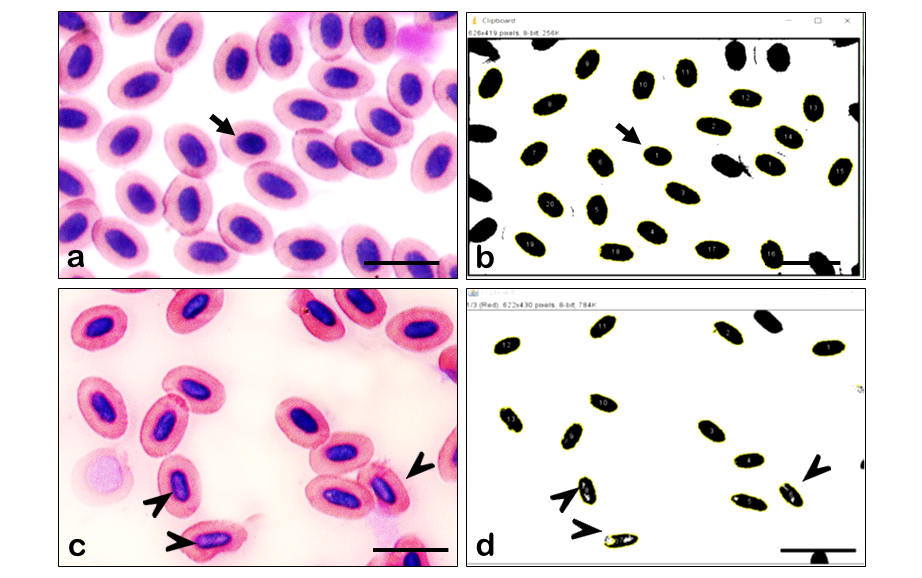

Supplement: Supplemental Information 3 — (A) a control sample; the arrow shows a regular shape and intact erythrocyte nucleus. (B) respective image in threshold adjusts; arrow shows the same nucleus from image a; yellow contour delimits the nuclear area for the pixel measure; (C) and (D) a Chloramine T (200 mg/L) group sample; note the abnormal shape and colorless nuclei (arrowhead) representative of irregular chromatin condensation. Bar scale = 10 µm. [file peerj-11-16452-s003.tif]
